# Photoelectrochemical properties of mesoporous NiO_*x*_ deposited on technical FTO via nanopowder sintering in conventional and plasma atmospheres

**DOI:** 10.1186/s40064-015-1265-3

**Published:** 2015-09-30

**Authors:** Muhammad Awais, Denis D. Dowling, Franco Decker, Danilo Dini

**Affiliations:** Department of Industrial Engineering, “King Abdulaziz” University, Rabigh, KSA; School of Chemical and Bioprocess Engineering, University College Dublin, Dublin, Ireland; Department of Chemistry, University of Rome “La Sapienza”, P.le Aldo Moro 5, 00185 Rome, Italy

**Keywords:** Nickel oxide, Nanopowder, *p*-type semiconductor, electrochemistry, Dye-sensitized solar cell, FTO, Solar conversion

## Abstract

Nanoporous nickel oxide (NiO_*x*_) has been deposited with two different procedures of sintering (CS and RDS). Both samples display solid state oxidation at about 3.1 V* vs* Li+/Li. Upon sensitization of CS/RDS NiO_*x*_ with erythrosine b (ERY), nickel oxide oxidation occurs at the same potential. Impedance spectroscopy revealed a higher charge transfer resistance for ERY-sensitized RDS NiO_*x*_ with respect to sensitized CS NiO_*x*_. This was due to the chemisorption of a larger amount of ERY on RDS with respect to CS NiO_*x*_. Upon illumination the photoinduced charge transfer between ERY layer and NiO_*x*_ could be observed only with oxidized CS. Photoelectrochemical effects of sensitized RDS NiO_*x*_ were evidenced upon oxide reduction. With the addition of iodine RDS NiOx electrodes could give the reduction iodine → iodide in addition to the reduction of RDS NiO_*x*_. p-type dye sensitized solar cells were assembled with RDS NiO_*x*_ photocathodes sensitized either by ERY or Fast Green. Resulting overall efficiencies ranged between 0.02 and 0.04 % upon irradiation with solar spectrum simulator (*I*_in_: 0.1 W cm^−2^).

## Background

Among semiconductors the class of metal oxides represents a particularly important group mainly because of the high chemical and physical stability in bulk (Ramanathan 2010) and nanocrystalline phases (Hagfeldt and Grätzel 1995). Other remarkable properties of semiconducting metal oxides are the display of mixed valence properties (cases of Nb, Ta, V, Mo, W or Ni oxides), (Cotton and Wilkinson 1988) and their electroactivity (Morrison 1980) that consists in the capability of being reversibly reduced and/or oxidized in the configuration of thin film (Yohe et al. 1968; Passerini and Scrosati 1994; Awais et al. 2013). Semiconducting oxides undergo also reversible solid state transitions of the type *semiconductor*-*to*-*metal* upon chemical/electrochemical doping (Dickens and Whittingham 1968; Masetti et al. 1995; Bueno et al. 2003). Such ensemble of features render metal oxides particularly interesting in those technologies (Smith and Granqvist 2010) based on the controlled modulation of the electrical, optical and dielectric properties. Within this class of functional materials a particularly important case is represented by nickel oxide the electrochemical (Awais et al. 2010, 2013; Marrani et al. 2014), electrochromic (Estrada et al. 1991; Decker et al. 1992) and photoelectrochemical (Awais et al. 2011, 2013; Sheehan et al. 2015) properties of which have been characterized and interpreted. In the most technologically relevant applications nickel oxide is in the configuration of thin film (thickness, *l*, 0.15 ≤ *l* ≤ 10 μm), and represents a defective system with non stoichiometric features (general formula: NiO_*x*_). The stoichiometric coefficient *x* is comprised in the range 1 < *x* < 1.5 depending on the relative concentration of Ni(III) centers with respect to Ni(II) centers (Mitoff 1961). The Ni(III) sites host the electronic vacancies (holes) that represent the positive charge carrier in neutral *p*-type NiO_*x*_. The concentration, mobility, diffusion properties and lifetime of the holes can be opportunely altered in NiO_*x*_ with the consequent modification of all electronic properties which depend on hole concentration (electrical conductivity, photoconductivity, redox properties, optical absorption, reflectance and dielectric constant) (Passerini and Scrosati 1994; Awais et al. 2010, 2011, 2013; Marrani et al. 2014; Estrada et al. 1991; Decker et al. 1992; Sheehan et al. 2015; Mitoff 1961; D’Amario et al. 2014; Boschloo and Hagfeldt 2001; He et al. 1999). One important application that utilizes NiO_*x*_ as functional material is the dye-sensitized solar cell (DSC) (O’Regan and Grätzel 1991). In these solar conversion devices NiO_*x*_ constitutes the photoelectroactive cathode (He et al. 2000). The main features of the NiO_*x*_ electrodes for *p*-type DSCs (*p*-DSCs) are the mesoporosity, the electrical connectivity between oxide nanoparticles, the large surface area, the optical neutrality and the capability of being efficaciously sensitized by NIR/vis light absorbers (He et al. 1999, 2000; Vera et al. 2005; Awais et al. 2014, 2015; Gibson et al. 2013). NiO_*x*_ for *p*-DSC purposes has been prepared in a variety of ways that differ for the nature of the precursors, procedure of preparation/deposition and thermal treatment (Dini et al. 2015). The photoelectrochemical properties of the resulting NiO_*x*_ films were dependent on the method of preparation and in a series of recent works we demonstrated the feasibility of a new scalable method of deposition and procedure of sintering for the preparation of NiO_*x*_ thin films which displayed very promising performances in *p*-DSC devices (Sheehan et al. 2015; Awais et al. 2014, 2015; Gibson et al. 2013). In fact, the combination of spray deposition and microwave plasma sintering (Awais et al. 2011) afforded NiO_*x*_ photocathodes that gave efficiencies in the order of 0.12 % when P1 was the sensitizer (Gibson et al. 2013). In these studies we defined also new indirect criteria for the evaluation of NiO_*x*_ electrode performance through the correlation between the redox properties of bare NiO_*x*_ in a three electrode cell and the photoelectrochemical properties of sensitized NiO_*x*_ in a two-electrode cell when a redox couple was present in the electrolyte (Sheehan et al. 2015; Awais et al. 2015; Gibson et al. 2013). In doing so, the electrochemical processes based on electroactive NiO_*x*_ and those based on the redox shuttle could be distinguished (Sheehan et al. 2015). Moreover, a mechanism of electrochemical switching could be verified with the occurrence of the recombination hole/reduced form of redox shuttle following the electrochemical injection of holes in NiO_*x*_ electrodes (Sheehan et al. 2015). Beside electrolyte composition, another factor controlling the electrochemical processes at NiO_*x*_ electrodes in DSCs is the nature of the supporting conductive substrate on which NiO_*x*_ coatings are deposited (Awais et al. 2015). In DSCs the supporting substrate must be transparent in order to warrant the transmission of light to the photoactive sensitized electrode. Another requirement of the transparent conductive substrate is its capability of transferring charge between NiO_*x*_ and the substrate itself for charge collection in the external circuit. The choices of the transparent conductive substrates for the support of NiO_*x*_ thin film electrodes for DSCs purposes are confined usually to indium-doped tin oxide (ITO) (Awais et al. 2013, 2015), fluorine-doped tin oxide (FTO) (Sheehan et al. 2015) and doped ZnO (Rousset et al. 2011). In the past, the influence of the nature of the substrate on the electrochemical properties of NiO_*x*_ for DSCs has been analyzed only in the case of ITO (Awais et al. 2013, 2015). Charge trapping phenomena with irreversible features were verified in ITO substrate when this was cathodically polarized (Awais et al. 2013, 2015). For this reason the choice of ITO as supporting substrate of NiO_*x*_ photocathodes for DSCs should be generally avoided or considered very carefully depending on the range of working potential. Since the study of the electrochemical properties of FTO substrate in relation with the redox and photoelectrochemical properties of NiO_*x*_ coating has never been attempted for *p*-DSCs purposes, the main motivation of the present work is to analyze for the first time the influence of the nature of FTO substrate (Granqvist 2007; Švegl et al. 2012; Nattestad et al. 2010; Powar et al. 2013) on the electrochemical properties of nanostructured NiO_*x*_ coatings deposited onto FTO (Gibson et al. 2013; Novelli et al. 2015; Bode et al. 1966; Lyons and Brandon 2008) via the two scalable methods of conventional sintering (CS) and microwave assisted plasma sintering (RDS) of preformed metal oxide nanoparticles (diameter <40 nm) (Awais et al. 2011; Sheehan et al. 2015). CS and RDS NiO_*x*_ samples were spray deposited onto transparent conducting oxide FTO to carry out the electrochemical and photoelectrochemical characterization of bare and dye-sensitised mesoporous NiO_*x*_ in non aqueous electrolyte. Both CS and RDS samples were sensitised with erythrosin B (ERY), i.e. a benchmark sensitizer for *p*-type DSCs (*p*-DSCs) (He et al. 1999, 2000; Vera et al. 2005; Awais et al. 2014, 2015; Gibson et al. 2013). The study has involved also the electrochemical characterization of uncoated FTO utilizing the same set of electrolytes used for the electrochemical/photoelectrochemical characterization of NiO_*x*_ coatings. In particular, we have considered the process of electrochemical oxidation of CS and RDS NiO_*x*_ in non aqueous electrolyte with various supporting electrolytes. We have also analysed the process of electrochemical reduction of nickel oxide samples in presence of iodine as redox species (O’Regan and Grätzel 1991; Boschloo and Hagfeldt 2009; Hagfeldt et al. 2010). The *p*-DSCs with the RDS NiO_*x*_ cathodes sensitized with ERY and Fast Green (Perera et al. 2003) colorants have been characterized.

## Experimental section

### Deposition of NiO_*x*_ coatings

Mesoporous NiO_*x*_ layers (thickness, *l*, varying in the range 0.4 ≤ *l* < 6 μm) were deposited by spraying onto FTO a suspension of NiO nanoparticles with diameter *d* < 50 nm (from Aldrich-Sigma) dispersed in alcoholic medium and then sintered with two different procedures: (1) conventional sintering (CS) (Awais et al. 2013); (2) rapid discharge sintering (RDS) (Awais et al. 2011). The maximum temperature of sintering was 450 °C in both CS and RDS procedures. Spray deposition of NiO_*x*_ nanoparticles was conducted on FTO substrate at room temperature. FTO-covered glass panels were purchased from Solaronix (TCO22-7). They were square-shaped and had a total area of 25 cm^2^. The description of the experimental setup for spraying the suspension of nickel oxide nanopowder has been reported by Halme et al. (2006). In the alcoholic dispersion the mass concentration of nickel oxide nanopowder was 20 mg mL^−1^ (medium: 2-propanol, from Aldrich). All chemicals were used as received.

### NiO_x_ sensitization

Prior the sensitization, CS and RDS NiO_*x*_ films on FTO were rinsed with ethanol and heated up to 100 °C for few minutes in order to remove possible adsorbed water. At that temperature they were immersed in the solution of ERY sensitizer [0.3 mM ERY (from Sigma-Aldrich) in ethanol (from Fisher)] for sixteen hours.

RDS sample has been sensitized also with Fast Green (Perera et al. 2003) upon immersion of the electrode in 0.2 mM Fast Green in acetonitrile (dipping time: 16 h). The dipping solutions were kept at ambient temperature. After removing the electrodes from the tincture solution, the colored electrodes were thoroughly washed with pure ethanol and pure acetonitrile to remove non chemisorbed dye molecules in the case of ERY and Fast Green sensitization, respectively.

### Electrochemical characterization of uncoated FTO

Similar to the electrochemical characterization we conducted previously on bare (Awais et al. 2013) uncoated FTO was employed as working electrode in a three-electrode cell and treated according to the procedure therein reported. Prior to any electrochemical test, bare FTO was kept in ultrasonic bath using isopropylic alcohol as solvent. The ultrasonic treatment lasts 30 min. Successively the ultrasonically cleaned FTO was dried in oven at 60 °C. After the cleaning treatment the FTO substrate was introduced in an Ar filled glove box. Uncoated FTO substrate was manipulated in the glove-box for cell assembly utilizing a three-electrode cell with Li rods (from Aldrich) as counter and reference electrodes (Masetti et al. 1995; Dini et al. 1996), and 0.7 M LiClO_4_ (from Aldrich) in anhydrous propylene carbonate (PC, from Fisher) as electrolyte. For the electrochemical characterization of FTO the potential values have been referred to the Li^+^/Li redox couple. The chemicals for the preparation of the electrolyte were used as received and were stored in an Ar filled glove-box (Innovative Technology, Newburyport, Massachusetts, USA). O_2_ and H_2_O content was below 10 ppm and 5 ppm, respectively. The electrochemical cell with FTO as working electrode was assembled in the Ar atmosphere of the glove-box. A supernatant Ar atmosphere was always maintained in the cell during the electrochemical experiments. The experiments of cyclic voltammetries and electrochemical impedance spectroscopy (EIS) were conducted with the CH electrochemical analyzer (model 604C, Austin, Texas, USA). EI spectra were recorded in the frequency range 5 × 10^−3^–1 × 10^5^ Hz in going from the highest to the lowest frequency.

### Electrochemical characterization of CS and RDS NiO_*x*_ films

The electrochemical characterization of the differently sintered NiO_*x*_ coatings was carried out in electrochemical cells with three-electrode configurations. Three-electrode cell configuration was the same as in case of bare FTO (vide supra) with working electrodes: glass/FTO/CS-NiO_*x*_, glass/FTO/RDS-NiO_*x*_, and the ERY-sensitized versions of both types of NiO_*x*_. In one type of three electrode configuration Li rods were used as counter and reference electrodes and the potential values were expressed relatively to the redox couple Li^+^/Li. The electrolyte had the same composition of the one used for the electrochemical tests of uncoated FTO.

In the second type of three-electrode configuration, the counter electrode was a Pt wire while reference was a standard calomel electrode (SCE). Nickel oxide based working electrodes were of CS and RDS types either in the bare or ERY-sensitized state. Electrolyte compositions were: (1) 0.5 M LiClO_4_ in PC; (2) 0.5 M TBAPF_6_ in PC (TBA: tetra-butyl-ammonium); (3) 0.5 M LiI in PC; (4) 0.5 M LiClO_4_, 0.05 M I_2_ in PC; (5) 0.5 M TBAPF_6_, 0.05 M I_2_ in PC. In some electrochemical experiments with the three-electrode cell we also used the non aqueous reference electrodes Ag/AgNO_3_ (Dini et al. 1999) and Ag/AgCl in anhydrous acetonitrile (Nattestad et al. 2010). In the two-electrode cell configuration, platinized FTO was used as counter electrode while the potential values were expressed as the difference of electrical potential built up by NiO_*x*_ working and Pt/FTO counter electrodes.

Cyclic voltammetries and EIS experiments with NiO_*x*_ working electrodes were conducted with the same set-up utilized for the analysis of the electrochemical properties of uncoated FTO (vide supra). In the experiments of photoelectrochemistry, the irradiation of bare and sensitized CS/RDS NiO_*x*_ electrodes was realized with a halogen lamp (power, *P* = 50 W), using an electrochemical cell provided with optical windows (optical window area: 25 cm^2^).

### p-DSC assembly

*p*-type DSCs with sensitized RDS NiO_*x*_ electrodes (photoelectroactive area: 5 × 5 mm^2^) were assembled by sealing the photocathodes and the counter electrode (platinum coated FTO) face-to-face in a sandwich configuration. Sealant was a 30 μm thick pre-cut Surlyn^®^ thermoplastic frame with interior area of 6 × 6 mm^2^ having the additional function of separator. The device was filled with the electrolyte 0.5 M I_2_, 0.05 M LiI in acetonitrile through a pre-drilled hole in the counter electrode under reduced pressure. The hole was sealed with Surlyn^®^ and a glass cover-slide. For the determination of the characteristic *JV* curves RDS NiO_*x*_ coatings were masked using a 4 × 4 mm opaque frame and in all cases the cells were illuminated from the side of the NiO_*x*_ working electrode (front illumination mode). The photovoltaic performance of NiO_*x*_ sensitized with ERY and Fast Green was evaluated using a solar simulator with AM1.5G spectral distribution.

## Results and discussion

### Electrochemical properties of FTO substrate

**Fig. 1:**
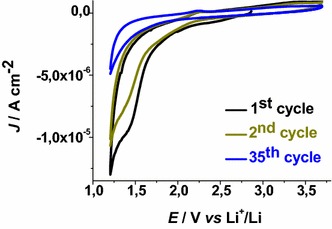
Evolution of the voltammogram of uncoated FTO. Electrolyte: 0.7 M LiClO_4_ in anhydrous propylene carbonate; counter electrode: Li; reference redox couple: Li^+^/Li; scan rate: 5 mV s^−1^. Open circuit voltage: 2.86 V* vs* Li^+^/Li

Technical FTO (95:5 ≤ SnO_2_:SnF_4_ < 99:1 molar ratio range; general formula: SnF_*x*_O_2_) with a sheet resistance of 7 Ω/□ and thickness <1 μm presents an irreversible wave of reduction between 1.1 and 2.2 V* vs* Li^+^/Li. The amplitude of this cathodic wave decreased upon continuous cycling (Fig. 1).

In analogy to ITO (Awais et al. 2013), such an electrochemical behavior is attributed to the initial irreversible uptake of lithium cations inside FTO according to the reaction:

1 $${\text{SnF}}_{x} {\text{O}}_{\text{y}} + z{\text{Li}}^{ + } + ze^{ - } \to {\text{Li}}_{z} {\text{SnF}}_{x} {\text{O}}_{\text{y}}$$

**Fig. 2:**
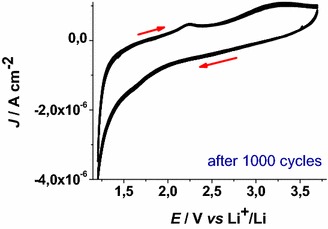
Stabilized cyclic voltammogram of FTO after prolonged cycling. Conditions as in Fig. 1. *Arrows* indicate the verse of scan

The electrochemically driven process of Eq. 1 leads to the permanent rearrangement of the FTO structure with the formation of Li_*z*_SnF_*x*_O_y_ (here indicated as lithiated FTO). Upon prolonged cycling the voltammogram of uncoated lithiated FTO stabilizes with the observation of the profile plotted in Fig. 2.

**Fig. 3:**
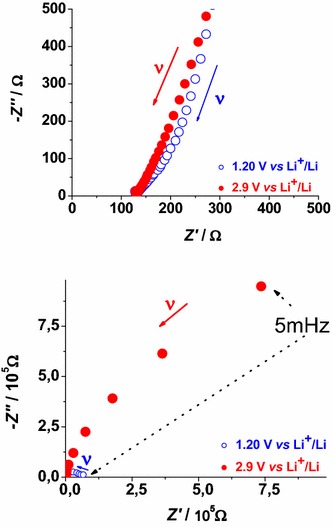
Nyquist plots in the high (*upper plot*) and low (*lower plot*) frequency range for FTO at 2.9 V* vs* Li^+^/Li and 1.2 V* vs* Li^+^/Li after stabilization of the electrochemical behavior (see Fig. 2). *Blue* and *red full* *arrows* indicate the verse of growing frequencies. *Black dotted arrows* indicate the point determined at the lowest applied frequency of 5 mHz

To investigate the changes of the electron transport properties in lithiated FTO, electrochemical impedance spectroscopy (EIS) has been utilized (Ho et al. 1980). The impedance spectra have been recorded when Li_*y*_SnF_*x*_O_2_ was polarized at 1.2 and 2.9 V* vs* Li^+^/Li (electrolyte: 0.7 M LiClO_4_ in anhydrous PC, Fig. 3). The electrochemical reaction of Li_*z*_SnF_*x*_O_y_ (Fig. 2) is the process of electrons uptake and release by Li_*y*_SnF_*x*_O_2_ respectively at 1.2 and 2.9 V* vs* Li^+^/Li with the accompanying uptake and release of lithium cations in order to preserve the electrical neutrality within Li_*w*+*z*_SnF_*x*_O_y_ (Eq. 2) (Cogan et al. 1985).

2 $${\text{Li}}_{Z} {\text{Sn}}_{x} {\text{O}}_{y} + w{\text{Li}}^{ + } + w\,e^{ - } \to {\text{Li}}_{w + z} {\text{SnF}}_{x} {\text{O}}_{y}$$

The EIS profile of Li_*z*_SnF_*x*_O_y_ recorded at 2.9 V* vs* Li^+^/Li presents an incomplete semicircle at low frequency whereas the signal of the lithiated system Li_*w*+*z*_SnF_*x*_O_y_ electrochemically formed at 1.2 V *vs* Li^+^/Li (Figs. 1, 2) is characterized by a completely defined semicircle in the same range of frequencies with amplitude smaller than that of Li_*z*_SnF_*x*_O_y_ (Fig. 3). Following lithiation (Eq. 1), the charge transport through the Li_*z*_SnF_*x*_O_y_ at 2.9 V and Li_*w*+*z*_SnF_*x*_O_y_ at 1.2 V layer is of mixed nature, i.e. both ionic and electronic (Chernyak et al. 1996). Anyhow, in the frequency range here analyzed there are no clear evidences of the diffusive characteristics usually associated with Warburg elements (Ho et al. 1980). We observe an increase of the through-layer resistance *R*_*l*_ (Ho et al. 1980) determined by the horizontal amplitude of the low frequency semicircle when the system passes from Li_*z*_SnF_*x*_O_y_ (56.7 kΩ) to Li_*w*+*z*_SnF_*x*_O_y_ (>100 kΩ). This trend indicates that the process of simultaneous lithium and electrons uptake in Li_*z*_SnF_*x*_O_y_ polarized at 1.2 V* vs* Li^+^/Li brings about the general increase of both electronic and ionic conductivity through the lithiated layer of FTO. The high frequency features associated with the capacitance built up by charge separation at the Li_*z*_SnF_*x*_O_y_ (or Li_*w*+*z*_SnF_*x*_O_y_)/electrolyte interface (double layer capacitance, *C*_DL_), and the parallel charge transfer resistance (*R*_*CT*_) of ionic insertion/extraction through the same interface at high frequency are not well defined. When compared to lithiated ITO (Awais et al. 2015) the corresponding values of the electrical parameters of lithiated FTO show a general diminution of the resistive terms.

Solid-state reduction of Li_*z*_SnF_*x*_O_y_ into Li_*w*+*z*_SnF_*x*_O_y_ represents a process of electrochemical *n*-doping with the formation of an accumulation layer which is expected to improves the surface-confined process of ionic charge transfer at the electrode/electrolyte interface. The resistive term *R*_*l*_ associated with the process of charge transfer through the layer is assumed to be inversely proportional to the number of mobile charge carriers (both ionic and electronic), which are present in the layer. This accounts for the decrease of *R*_*l*_ in passing from 2.9 to 1.2 V* vs* Li^+^/Li since the number of mobile charge carriers of both nature increases within lithiated FTO upon occurrence of the solid state reduction reported in Eq. 2.

### Electrochemical properties of RDS and CS NiO_x_ samples

**Fig. 4:**
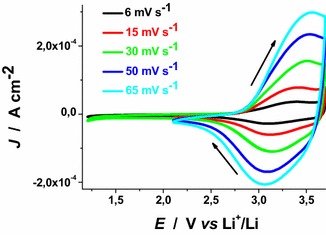
Cyclic voltammetries of CS NiO_*x*_ on FTO substrate at different scan rates. Open circuit potential (OCV): 3.07 V* vs* Li^+^/Li. (*J* current density, *E* applied potential). Electrolyte as in Fig. 1. *Arrows* indicate the verse of scan

CS NiO_*x*_ sample presents a quasi-reversible oxidation peak centred approximately at 3.40 V* vs* Li^+^/Li when scan rate is 6 mV s^−1^ (Fig. 4). Such a peak presents broad features and refers to the conversion of Ni(II) into Ni(III) within the oxide film (Boschloo and Hagfeldt 2001).

**Fig. 5:**
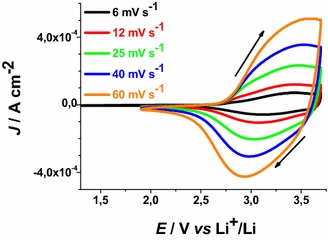
Cyclic voltammetries of RDS-NiO_x_ on FTO substrate at different scan rates. OCV: 3.24 V* vs* Li^+^/Li. Electrolyte as in Fig. 1. *Arrows* indicate the verse of scan

The onset of CS oxidation is about 2.85 V* vs* Li^+^/Li. This indicates that the pristine film of CS contains a fraction of Ni(III) sites the open circuit voltage (OCV) being 3.07 V* vs* Li^+^/Li. This has been further confirmed by XPS data that present signals associated to the presence of Ni(III) in pristine mesoporous NiO_*x*_ (Marrani et al. 2014; Gibson et al. 2013). Analogous considerations hold in the case of RDS nickel oxide samples (Fig. 5). RDS NiO_*x*_ is characterized by the onset of oxidation at about 2.75 V* vs* Li^+^/Li, and by an OCV value of 3.24 V* vs* Li^+^/Li.

**Fig. 6:**
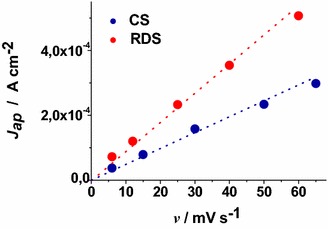
Scan rate dependence of the anodic peaks of CS-NiO_*x*_ and RDS-NiO_*x*_ with the same thickness and deposited onto FTO substrate. Data are taken from the cyclic voltammetries of Figs. 4 and 5 (*J*_*ap*_ current density in correspondence of the anodic peak, *v* scan rate)

When the scan rate dependence of the oxidation peaks of CS NiO_*x*_ and RDS NiO_*x*_ is analysed (Fig. 6), a linear relationship between peak height and scan rate is found. This corresponds to the occurrence of a surface confined redox process (Bard and Faulkner 2001) the rate of which does not depend on the diffusion of charge carriers or mass transfer processes.

**Fig. 7:**
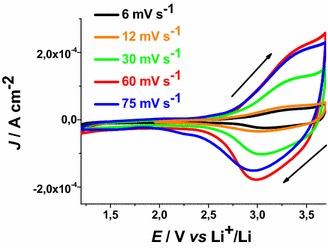
Dark cyclic voltammetries of ERY-sensitised CS-NiO_*x*_ on FTO substrate at different scan rates. *Arrows* indicate the verse of scan

**Fig. 8:**
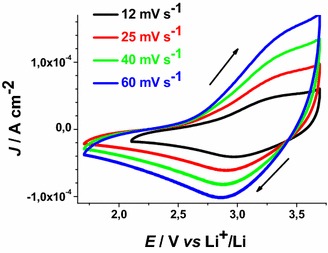
Dark cyclic voltammetries of ERY-sensitised RDS-NiO_*x*_ on FTO substrate at different scan rates. *Arrows* indicate the verse of scan

These results have been determined in anhydrous electrolyte and are consistent with the data reported by Boschloo et al. (2001) who characterized sol–gel NiO_*x*_ films in aqueous and non aqueous electrolytes. The comparative analysis of the electrochemical properties of CS NiO_*x*_ and RDS NiO_*x*_ shows that RDS sample in the pristine state contains a larger amount of Ni(III) sites with respect to CS sample due to the larger OCV of the RDS oxide. Moreover, the larger current densities (Figs. 4, 5), and the larger slope of the curve *J*_*ap*_*vsv* for RDS with respect to CS NiO_*x*_ (Fig. 6), indicate that the RDS oxide has a larger density of surface-confined electroactive sites in comparison to the CS sample. This consideration is valid when the process of NiO_*x*_ oxidation is considered taking into account that the two samples have the same film thickness. This is similar to what we found with CS and RDS NiO_*x*_ when the electrolyte was aqueous and contained a phosphate buffer (Gibson et al. 2013). Dye-sensitisation of both NiO_*x*_ samples with ERY generally leads to a decrease of the dark current densities (Figs. 7, 8), with respect to the corresponding curves determined with the bare nickel oxides (Figs. 4, 5).

**Fig. 9:**
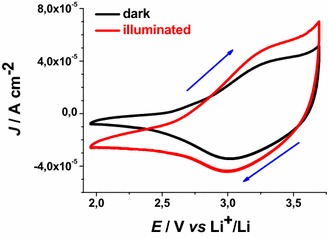
Cyclic voltammetry of ERY-sensitised CS-NiO_x_ deposited on FTO substrate (scan rate: 12 mV s^−1^). OCV: 3.13(dark)/3.17(illuminated) V* vs* Li^+^/Li. Radiation source was a halogen white lamp with total intensity of 22 W cm^−2^. *Arrows* indicate the verse of scan

**Fig. 10:**
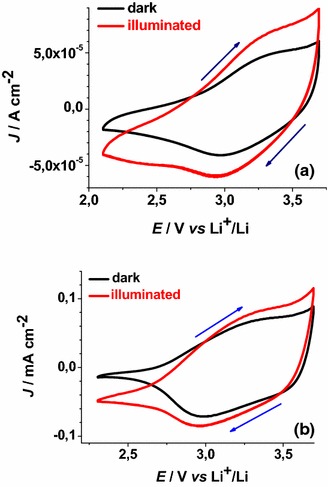
Cyclic voltammetry of ERY-sensitised RDS-NiO_x_ deposited on FTO substrate [scan rate: **a** 12 and **b** 25 mV s^−1^]. OCV: 3.21(dark)/3.24(illuminated) V* vs* Li^+^/Li. Radiation source was a halogen white lamp with total intensity of 25 W cm^−2^. *Arrows* indicate the verse of scan

**Fig. 11:**
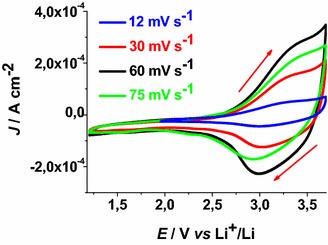
Cyclic voltammetries of illuminated ERY-sensitised CS-NiO_x_ on FTO substrate at different scan rates (incident intensity: 22 W cm^−2^). *Arrows* indicate the verse of scan

**Fig. 12:**
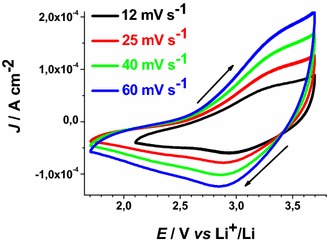
Cyclic voltammetries of illuminated ERY-sensitised RDS-NiO_x_ on FTO substrate at different scan rates (incident intensity: 25 W cm^−2^). *Arrows* indicate the verse of scan

No additional current peaks related to ERY electrochemistry are found within the experimental range of applied potential. Under these circumstances the layer of ERY behaves as a passivating agent towards the process of ionic charge transport through ERY-NiO_*x*_/electrolyte interface. Moreover, ERY layer is electrochemically inert due to the absence of any faradic process associated to ERY. White light illumination of the dye-sensitised oxide samples produces several effects: a positive photopotential, an increase of the oxidation current density, and the negative shift of the current baseline when no redox processes occur (Figs. 9, 10). This combination of facts is due to the photogeneration of positive charge carriers (Gerischer and Willig 1976) in dye-sensitised NiO_*x*_ when visible light is absorbed by the ERY layer (He et al. 1999, 2000; Vera et al. 2005). Cyclic voltammetries of ERY-sensitised RDS and ERY-sensitised CS NiO_*x*_ have been carried out at different scan rates under white light illumination (Figs. 11, 12).

**Fig. 13:**
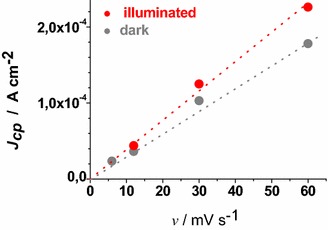
Scan rate dependence of the cathodic peaks of ERY-CS NiO_x_ in the dark and under illumination. Data are taken from the cyclic voltammetries of Figs. 7 and 11 (*J*_*cp*_ : current density in correspondence of the cathodic peak)

**Fig. 14:**
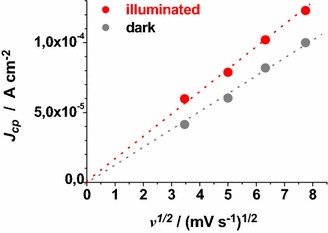
Scan rate dependence of the cathodic peaks of ERY-RDS NiO_x_ in the dark and under illumination. Data are taken from the voltammograms of Figs. 8 and 12

ERY-sensitised samples of RDS and CS present a markedly different behaviour when the dependence of the current density peaks on scan rate was analysed (Figs. 13, 14).

The cathodic peak referring to the process Ni(III) → Ni(II) has been considered for analysis because of its better definition with respect to the associated anodic peak in the same voltammogram (Figs. 13, 14). Like unmodified bare CS, the electrochemical oxidation of ERY-sensitized CS NiO_*x*_ presents the typical features of a surface confined redox process (Fig. 13), whereas the oxidation of ERY-sensitized RDS NiO_*x*_ presents a linear dependence of the current density peak on the square root of scan rate (Fig. 14). The latter feature of the RDS sample indicates that its ERY-sensitised version undergoes an oxidation process which is diffusion controlled rather than surface confined (Bard and Faulkner 2001). A possible explanation for such a difference could be the growth of a thin film of ERY on the surface of RDS NiO_*x*_, whereas sensitisation of CS NiO_*x*_ produces solely a monolayer (or sub-monolayer)of dye on the surface of CS NiO_*x*_. This is explainable if we assume that the number of anchored molecules of ERY is directly proportional to the number of the electroactive surface sites which constitute the actual sites of dye anchoring on the surface of nickel oxide. Pristine RDS sample is characterised by the presence of a larger number of Ni(III) surface sites states in comparison to CS NiO_*x*_ as proved by XPS experiments (Gibson et al. 2013) and by the electrochemical measurements here reported (vide supra). As a consequence of that, it is expected that a larger number of dye molecules gets anchored on the surface of RDS with respect to CS.

**Fig. 15:**
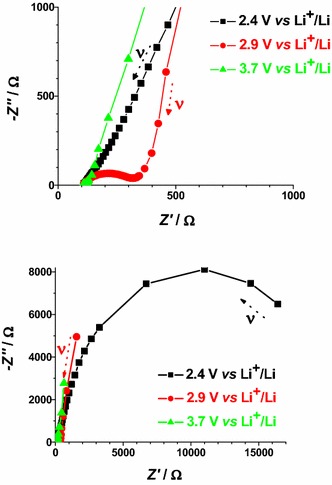
Nyquist plots in the high (*upper plot*) and low (*lower plot*) frequency range for RDS NiO_*x*_ at 2.4, 2.9 and 3.7 V* vs* Li^+^/Li. *Black* and *red arrows* indicate the verse of growing frequencies

**Fig. 16:**
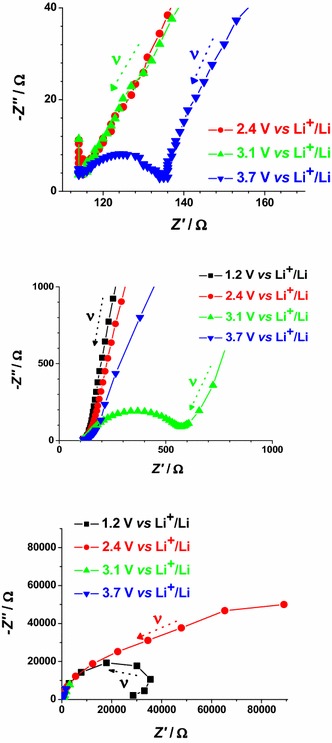
Nyquist plots in the high (*upper plot*), medium (*center plot*), and low (*lower plot*) frequency range for CS NiO_*x*_ at 1.2, 2.4, 3.1 and 3.7 V* vs* Li^+^/Li. *Arrows* indicate the verse of growing frequencies for the different profiles

**Fig. 17:**
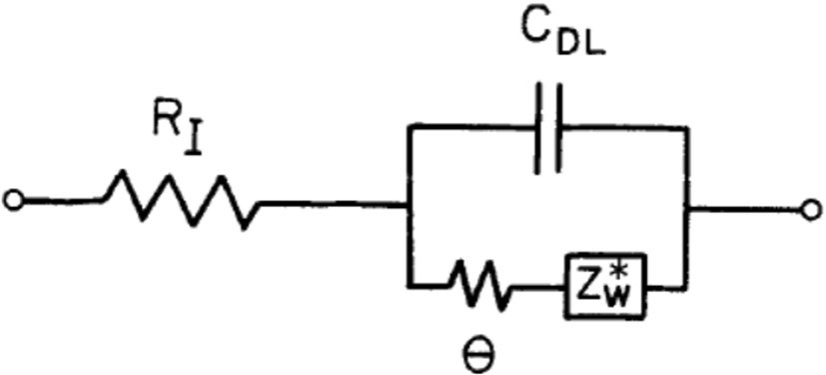
Equivalent circtuit of Randles type (adapted from Ho et al. 1980). For symbols explanation, please, refer to Halme et al. (2006)

The electrochemical impedance spectra of CS- and RDS-NiO_*x*_ have been recorded at different applied potential values. The chosen values of polarization correspond to different states of oxidation of the NiO_*x*_ samples (Figs. 4, 5). The impedance spectra reflect the changes of the electrical transport properties that NiO_*x*_ undergoes when the oxide alters the state of oxidation (Decker et al. 1992). Data are presented in Figs. 15 and 16. For semiconducting metal oxides possessing intercalation properties, the model which is usually adopted for the interpretation of their impedance spectra is the one of Ho et al. (1980) who proposed the Randles circuit (Fig. 17). This model accounts for the phenomena related with the charge transfer across the interface NiO_*x*_ electrode/electrolyte through the resistive term *θ* (charge transfer resistance through the interface), and the capacitive term *C*_DL_ (double layer capacitance). Moreover it accounts also for the transport properties associated with the diffusive motion of the charge carriers through the electrode itself (term *Z*_W_* related to the diffusion of electroactive species) (Ho et al. 1980).

**Fig. 18:**
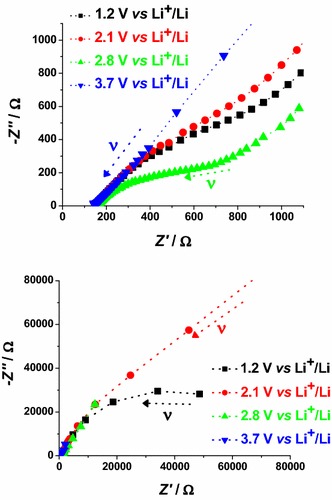
Nyquist plots in the high (*upper plot*), and low (*lower plot*) frequency range for ERY sensitized CS NiO_*x*_ at 1.2, 2.1, 2.8 and 3.7 V* vs* Li^+^/Li. *Arrows* indicate the verse of growing frequencies for the different profiles

**Fig. 19:**
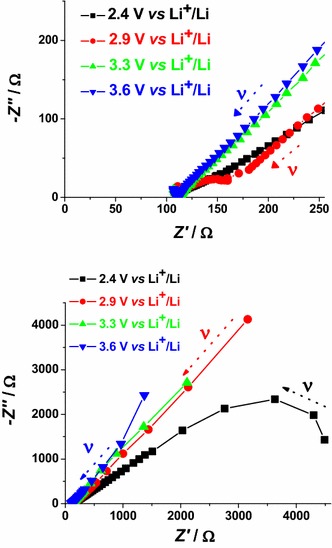
Nyquist plots in the high (*upper plot*), and low (*lower plot*) frequency range for ERY sensitized RDS NiO_*x*_ at 2.4, 2.9, 3.3 and 3.6 V* vs* Li^+^/Li. *Arrows* indicate the verse of growing frequencies for the different profiles

**Fig. 20:**
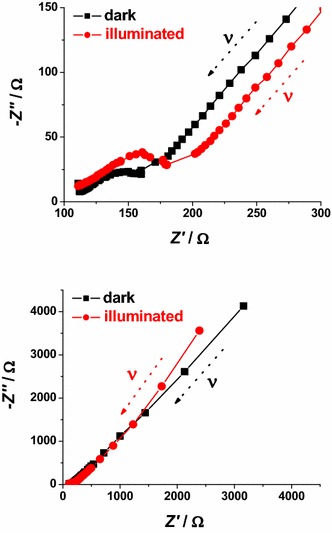
Effect of light on the Nyquist plots of ERY sensitized RDS NiO_*x*_ polarized at 2.9 V* vs* Li^+^/Li. High (*upper plot*), and low (*lower plot*) frequency range. *Arrows* indicate the verse of growing frequencies for the different profiles. White light intensity: 25 W cm^−2^

**Fig. 21:**
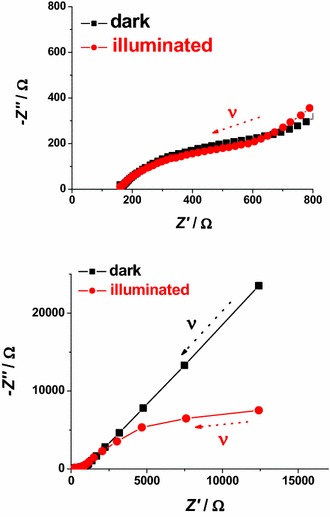
Effect of light on the Nyquist plots of ERY sensitized CS NiO_*x*_ polarized at 2.8 V *vs* Li^+^/Li. High (*upper plot*), and low (*lower plot*) frequency range. *Arrows* indicate the verse of growing frequencies for the different profiles. White light intensity: 22 W cm^−2^

In case of bare NiO_*x*_ the main effect of polarization is the alteration of the interfacial parameters. We can identify states of polarization of NiO_*x*_ with low interfacial resistance when NiO_*x*_ is polarized at *E*_appl_ > 2.9 V* vs* Li^+^/Li. In the oxidized state NiO_*x*_ presents a well defined semicircle associated with interfacial processes (high frequency signal). This signal dose not overlap with other impedance spectral features, and is distinct from the diffusive processes associated to the motion of the charges electrochemically introduced in NiO_*x*_. These processes are controlled by diffusion phenomena and characterize the EIS signal at the lower frequencies. When polarization is applied at *E*_appl_ ≤ 2.4 V* vs* Li^+^/Li, NiO_*x*_ is either in the neutral or in a partially reduced state. Under these circumstances, NiO_*x*_ presents a very large interfacial resistance (broad and incomplete semicircle in the high frequencies range) no matter of the modality of preparation and sintering (Figs. 15, 16). Upon sensitization with ERY, the dark electrochemical impedance spectra of CS samples polarized at different values of *E*_appl_ (Fig. 18) show a trend analogous to that of the bare CS oxide (Fig. 16). This similarity indicates that the dye layer chemisorbed on the oxide does not form actually an electrochemical interface with the underlying NiO_*x*_ substrate. In the impedance spectra of ERY sensitized RDS samples (Fig. 19) there is no clear distinction between interfacial processes with fast kinetic features and the slowest diffusive phenomena in comparison to the corresponding bare RDS oxide (Fig. 15). This could be due to the larger amount of adsorbed dye in RDS samples with respect to the corresponding CS sample with the same thickness (vide supra) (Awais et al. 2013; Gibson et al. 2013). As previously discussed, the formation of a thick layer of ERY on RDS NiO_*x*_ sample creates a barrier against the charge transfer to the underlying oxide. The effect of light on the transport properties of ERY sensitized RDS is very weak (Fig. 20). This is another evidence of the lack of charge transfer processes between RDS and the layer of ERY when the latter does not have the features of a monolayer but behaves as a passivation layer with finite thickness (Awais et al. 2014). For ERY modified CS NiO_*x*_ we verified a photoinduced process of charge transfer between ERY layer and CS NiO_*x*_ substrate through the observation of a second broad incomplete semicircle at lower frequencies following the high frequency signal (Fig. 21).

### Photoelectrochemical properties of RDS NiO_x_ with different electrolytes

In this section we present a series of voltammetry experiments conducted in anhydrous propylene carbonate with different supporting electrolytes (LiClO_4_, TBAPF_6_) at the concentration of 0.5 mol per litre. The working electrode was a thin film of RDS-NiO_*x*_ (either bare or in the ERY sensitized state), a Pt wire was the counter electrode, and a standard calomel electrode (SCE) was taken as reference electrode. The very fast kinetics of electron transfer at Pt electrodes renders the kinetics of the overall electrochemical reaction controlled exclusively by the working electrode made of semiconducting NiO_*x*_. The current profiles have been determined either in the dark or under illumination with AM 1.5 Solar Simulator.

**Fig. 22:**
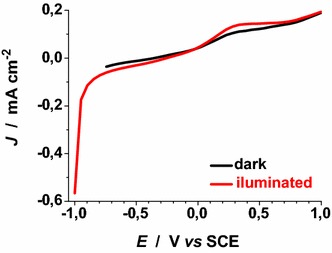
Effect of light on the voltammogram of bare RDS NiO_*x*_. Electrolyte: 0.5 M LiClO_4_ in propylene carbonate. Electrode is illuminated with white light (intensity: 25 W cm^−2^)

**Fig. 23:**
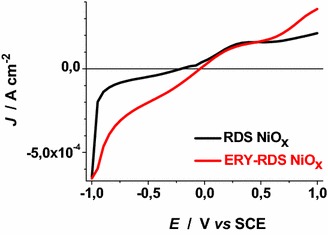
Effect of ERY sensitization on the voltammogram of RDS NiO_*x*_ under illumination. Electrolyte: 0.5 M LiClO_4_ in propylene carbonate. Electrode is illuminated with white light (intensity: 25 W cm^−2^)

**Fig. 24:**
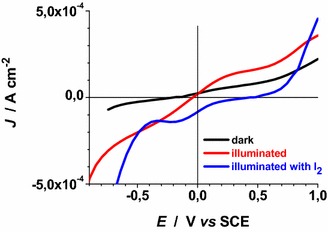
Comparison of voltammograms of ERY-RDS NiO_*x*_ in the dark (*black line*), illuminated with AM 1.5 Solar Simulator with redox species (I_2_) in the electrolyte (*green line*), illuminated with AM 1.5 Solar Simulator without redox species (*red line*). Electrolytes: 0.5 M LiClO_4_ in propylene carbonate (*red* and *black curve*); 0.5 M LiClO_4_ + 0.05 M I_2_ in propylene carbonate (*green curve*)

**Fig. 25:**
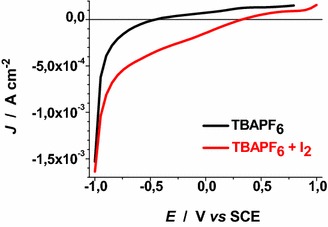
Dark voltammograms of bare RDS NiO_*x*_. Electrolytes: 0.5 M TBAPF_6_ and 0.5 M TBAPF_6_+ 0.05 M I_2_ in propylene carbonate

**Fig. 26:**
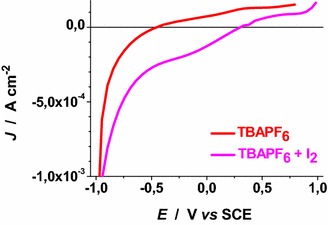
Voltammograms of bare RDS NiO_*x*_ illuminated with AM 1.5 Solar Simulator. Electrolytes as in Fig. 25

**Fig. 27:**
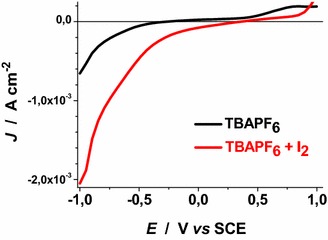
Dark voltammograms of ERY sensitized RDS NiO_*x*_. Electrolytes: 0.5 M TBAPF_6_ and 0.5 M TBAPF_6_ + 0.05 M I_2_ in propylene carbonate

**Fig. 28:**
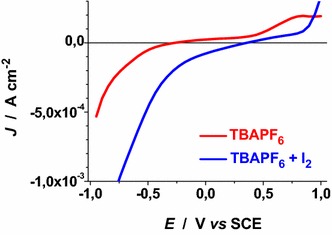
Voltammograms of ERY sensitized RDS NiO_*x*_ illuminated with AM 1.5 Solar Simulator. Electrolytes as in Fig. 27

Voltammograms of Figs. 22, 23, 24, 25, 26, 27, 28 have been determined in a single scan in going from +1 to −1 V* vs* SCE at the span rate of 20 mV s^−1^. In the potential range here examined RDS NiO_*x*_ can undergo both oxidation and reduction processes (Awais et al. 2013). Moreover, we have considered also electrolytes containing iodine as electroactive species. This is because iodine is one constituent of the main redox couple utilized in DSCs applications (Boschloo and Hagfeldt 2009). The main motivation of this study is the distinction between the electrochemical/photoelectrochemical signals generated by the electroactivity of RDS NiO_*x*_ deposited onto FTO, from those produced by the redox species of *p*-DSCs (Dini et al. 2015).

In Fig. 22 the effect of light on the electrochemical processes of bare NiO_*x*_ (oxidation at about 0.25 V* vs* SCE, and reduction (Passerini and Scrosati 1994) at −0.9 V* vs* SCE) is shown. We observe that light does not alter considerably the kinetics of bare NiO_*x*_ based processes when LiClO_4_ is the supporting electrolyte. Upon sensitization with ERY there is the introduction of a broad peak of reduction at applied potential values comprised between 0 and −0.5 V* vs* SCE (Fig. 23). This signal has to be ascribed to the electroactivity of the ERY film (Awais et al. 2013) in the configuration of thick layer when it is anchored on RDS-NiO_*x*_ (vide supra). The main consequence of that is the capability of ERY film of displaying capacitive effects (Awais et al. 2013). When ERY-RDS NiO_*x*_ is illuminated the addition of iodine I_2_ at the concentration of 0.05 M introduces a new broad peak of reduction within the potential range −0.4 < *E*_appl_ < 0.15 V* vs* SCE. This additional peak is ascribed to the reduction of I_2_ (Fig. 24).

Voltammograms of bare RDS-NiO_*x*_ with TBAPF_6_ as supporting electrolyte in dark and illuminated conditions are shown in Figs. 25 and 26, respectively. Like the case of LiClO_4_ based electrolytes (Figs. 23, 24), the presence of I_2_ introduces a broad peak of reduction the onset of which is located at about 0.3 V* vs* SCE. The condition of illumination does not seem to affect considerably the kinetic of the process of I_2_ reduction at bare RDS-NiO_*x*_ when TBAPF_6_ is the supporting electrolyte. Upon ERY sensitization of RDS-NiO_*x*_ the voltammograms recorded in dark (Fig. 27) and illuminated (Fig. 28) conditions present features that resemble the profiles of bare NiO_*x*_ when the electrolyte is the same (Figs. 25, 26). Than main difference between bare and ERY-sensitized RDS-NiO_*x*_ is the increase of the cathodic current associated with the process of I_2_ reduction when ERY-sensitized RDS-NiO_*x*_ is illuminated. Despite the finite thickness of the sensitizer layer, the system ERY-sensitized RDS-NiO_*x*_ is capable to photoreduce I_2_ in an effective way.

### Characteristic curves of the p-DSCs with RDS NiO_x_ photocathodes

**Fig. 29:**
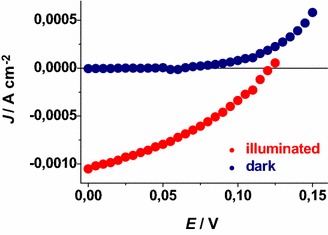
*JV* characteristic curve of the *p*-DSC with RDS NiO_*x*_ deposited onto FTO as photoelectroactive cathode. NiO_*x*_ film thickness: 2.5 ± 0.3 μm; dye-sensitizer: ERY; solar simulator intensity: 0.1 W cm^−2^. *η* = 0.045 %; *V*_OC_ = 0.120 V; *J*_SC_ = 1.05 mA cm^−2^; FF = 36.0 %

**Fig. 30:**
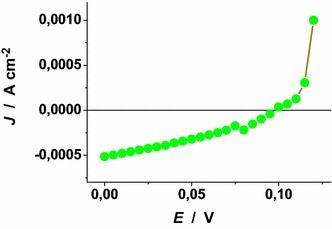
*JV* characteristic curve of the *p*-DSC with RDS NiO_*x*_ deposited onto FTO as photoelectroactive cathode. NiO_*x*_ film thickness: 1.5 ± 0.3 μm; dye-sensitizer: fast green; solar simulator intensity: 0.1 W cm^−2^. *η* = 0.018 %; *V*_OC_ = 0.098 V; *J*_SC_ = 0.51 mA cm^−2^; FF = 36.7 %

The *JV* curves of the *p*-DSCs with ERY and Fast Green sensitized RDS NiO_*x*_ photocathodes have been determined upon irradiation with solar light simulator (Figs. 29, 30). The overall conversion efficiency was higher for the photoelectrochemical cells with ERY sensitizer with respect to the devices utilizing Fast Green dye (*η*: 0.045* vs* 0.018 %). Moreover, the values of short circuit current density (*J*_SC_) and open circuit potential (*V*_OC_) were systematically higher for the devices with ERY sensitizer in comparison to those using Fast Green (*J*_SC_: 1.05* vs* 0.51 mA cm^−2^; *V*_OC_: 0.120* vs* 0.098 V). In terms of cyclic stability the *p*-DSCs with ERY reproduced at least 70 consecutive *JV* cycles whereas the Fast Green based devices presented repeatability of their photoelectrochemical behaviour in at least 30 consecutive cycles. In this context the reproducibility of the *p*-DSC is defined as the number of consecutive *JV* cycles during which the cell displays a diminution of the three relevant cell parameters *J*_SC_, *V*_OC_ and *η* not larger than 5 % of their maximum values. The three relevant parameters *J*_SC_, *V*_OC_ and *η* determined with the *p*-DSCs considered here were comparatively larger than the ones obtained with differently prepared NiO_*x*_ photoelectrodes when the same set of dyes was utilized and nickel oxide film thickness was the same (Awais et al. 2014; Dini et al. 2015; Perera et al. 2005).

## Conclusions

The electroactivity of two differently prepared NiO_*x*_ samples (CS and RDS) in the configuration of thin films has been verified for the solid state oxidation reaction Ni(II) → Ni(III) and the reduction process NiO_*x*_ + *n e*^−^ + *n* Li^+^ → Li_*n*_NiO_*x*_. CS and RDS NiO_*x*_ showed similar electrochemical behaviours with RDS displaying larger current densities of oxidation and possessing a higher number of Ni(III) surface sites with respect to CS. Both bare oxide samples undergo surface-confined oxidation as proved by the linear relationship between current peaks and scan rate. Upon sensitisation with ERY colorant Ni(II) oxidation is no longer surface–confined but becomes diffusion controlled in the sole case of RDS. This has been explained in terms of a larger surface concentration of dye molecules on the RDS surface with respect to CS, with the formation of a finite thickness film of dye (and not a monolayer) onto sensitized RDS. This conclusion has been confirmed also through the analysis of the electrochemical impedance spectra. The electrochemical properties of RDS NiO_*x*_ have been characterized in presence of the redox species I_2_ in the electrolyte. In this last series of experiments the potential was applied within the typical range of iodine reduction. We could distinguish the redox processes based on the solid state electroactivity of NiO_*x*_ and those based on iodine electrolyte. Moreover, we found out that the photocurrent of ERY-sensitised RDS was cathodic when iodine represented the redox active species. This confirmed the *p*-type character of the ERY sensitised oxide. RDS samples were also employed as photoactive cathodes in *p*-DSC devices. The comparison of the *p*-DSCs characteristic curves with ERY and Fast Green sensitizers showed a more effective photoelectrochemical performance of the device with ERY dye-sensitizer in terms of conversion efficiency and cyclability.
